# High-Speed Fabrication of Clear Transparent Cellulose Nanopaper by Applying Humidity-Controlled Multi-Stage Drying Method

**DOI:** 10.3390/nano10112194

**Published:** 2020-11-04

**Authors:** Chenyang Li, Takaaki Kasuga, Kojiro Uetani, Hirotaka Koga, Masaya Nogi

**Affiliations:** The Institute of Scientific and Industrial Research, Osaka University, 8-1 Mihogaoka, Ibaraki, Osaka 567-0047, Japan; lichenyang@eco.sanken.osaka-u.ac.jp (C.L.); tkasuga@eco.sanken.osaka-u.ac.jp (T.K.); uetani@eco.sanken.osaka-u.ac.jp (K.U.); hkoga@eco.sanken.osaka-u.ac.jp (H.K.)

**Keywords:** cellulose nanofibers, transparent nanopaper, fast-drying fabrication, relative humidity control, multi-stage drying

## Abstract

As a renewable nanomaterial, transparent nanopaper is one of the promising materials for electronic devices. Although conventional evaporation drying method endows nanopaper with superior optical properties, the long fabrication time limits its widely use. In this work, we propose a multi-stage drying method to achieve high-speed fabrication of clear transparent nanopaper. Drying experiments reveal that nanopaper’s drying process can be separated into two periods. For the conventional single-stage evaporation drying, the drying condition is kept the same. In our newly proposed multi-stage drying, the relative humidity (RH), which is the key parameter for both drying time and haze, is set differently during these two periods. Applying this method in a humidity-controllable environmental chamber, the drying time can be shortened by 35% (from 11.7 h to 7.6 h) while maintaining the same haze level as that from single-stage drying. For a conventional humidity-uncontrollable oven, a special air flow system is added. The air flow system enables decrease of RH by removing water vapor at the water/air interface during the earlier period, thus fabricating clear transparent nanopaper in a relatively short time. Therefore, this humidity-controlled multi-stage drying method will help reduce the manufacturing time and encourage the widespread use of future nanopaper-based flexible electronics.

## 1. Introduction

Cellulose nanopaper is composed of only cellulose nanofibers, which are mainly generated from wood [1,2]. The general procedure to produce cellulose nanopaper is as follows. First, the wood chips are purified and disintegrated into micro-sized cellulose pulp fibers, and the pulp fibers are then further mechanically nanofibrillated in water to obtain a cellulose nanofiber dispersion. Finally, the cellulose nanofiber dispersion is dried using evaporation or vacuum-filtration-assisted drying to prepare optically transparent cellulose nanopaper [3]. The type of cellulose nanofiber dispersion is the key factor controlling the transparency of the cellulose nanopaper. The dispersion containing coarse nanofibers of microfibrillated cellulose can be dried within 12 h by heating after filtration; however, the obtained nanopaper will be translucent because of its low sheet density [4,5,6]. In contrast, if the dispersion only contains fine nanofibers of cellulose microfibrils (2–4-nm wide) or bundles of cellulose microfibrils (approximately 15-nm wide), a long drying time ranging from overnight to a few days is required [1,2,3,7,8,9,10,11,12,13,14,15,16,17,18,19]. For the case in which the dispersion only contains fine nanofibers, the obtained nanopaper will be clear transparent with low haze [7]. This outcome is achieved because the fine nanofibers are densely packed, resulting in the absence of cavities and thus preventing light from scattering inside the sheet [1].

Because the properties of translucent nanopaper prepared in a short drying time are equivalent to those of traditional opaque paper, their applications are limited to layer coatings or additives for traditional paper. In contrast, transparent cellulose nanopaper, which requires a long drying time, offers various improved properties relative to those of translucent nanopaper, traditional paper, and certain polymer films. These improved properties include clear transparency, surface smoothness, high dielectric constant, and electrical insulation [20,21,22]. In the near future, flexible electronics will be applied owing to their excellent electrical and optical performance. Compared with traditional fossil-based materials, such as plastic films, transparent cellulose nanopaper is one of the best candidates for future flexible electronic substrates including solar cells, memory, transparent electrodes, sensors, and transistors [12,19,22,23,24]. Although various drying processes were developed over the past few years, rapid drying processes do not yet meet the desired standards. For the realization of these future applications, an in-depth understanding of the correlation between the moisture evaporation of dispersions and the cellulose nanopaper microstructure is necessary.

The drying mechanism of transparent cellulose nanopaper from fine cellulose nanofiber dispersions was recently investigated. Traditionally, the two main drying routes were vacuum-filtration-assisted drying, in which the dispersion is vacuum filtrated and the obtained wet sheet is heated using a hot press or oven drying [1,2,3,7], and evaporation drying, in which the dispersion is dropped onto a flat substrate and then heated via oven drying [3,8,9,10,11,12,13,14,15,16,17,18,19]. Evaporation drying produces cellulose nanopaper with relatively superior mechanical properties, optical transparency, and gas barrier properties as well as high heat-transfer properties and electrical resistivity owing to the nematic-ordered and densely packed arrangement of nanofibers [3]. Although evaporation drying resulted in more desirable properties, it comes at the expense of drying time. Moreover, during evaporation drying, high humidity improves the transparency of the nanopaper; however, it also prolongs the drying time [18].

In this work, we report that the drying time of cellulose nanopaper can be reduced while maintaining its clear transparency by controlling the drying condition. The essential requirement for clear transparency is the use of a fine cellulose nanofiber dispersion. As a typical type of fine cellulose nanofibers of cellulose microfibrils that can be easy to disperse in water homogeneously, TEMPO (2,2,6,6-tetramethylpiperidine-1-oxyl radical)-oxidized cellulose nanofibers (3–5-nm wide) [25,26] were used as the starting materials for the transparent cellulose nanopaper. The transparent cellulose nanopaper was prepared using evaporation drying. During the drying process, the dispersion weight change was monitored to understand the evaporation mechanism. After drying, the haze and density of the cellulose nanopaper were measured to investigate its microstructure. On the basis of our findings, we proposed a humidity-controlled multi-stage drying method for transparent cellulose nanopaper to reduce the drying time while maintaining its clear transparency.

## 2. Materials and Methods

### 2.1. Cellulose Nanofiber Dispersion

The TEMPO-oxidized cellulose nanofiber dispersion (RHEOCRYSTA I-2SX, DKS Co., Ltd., Kyoto, Japan) was used as a starting material. The 2 wt % RHEOCRYSTA I-2SX dispersion was diluted to 0.5 wt % and then stirred for 30 min. To remove the nanofiber aggregations, the diluted dispersion was passed 10 times at 245 MPa through a high-pressure water-jet system (Star Burst, HJP-25008, Sugino Machine Co., Ltd., Toyama, Japan) equipped with a ball-collision chamber. The obtained 0.45 wt % dispersion was then degassed using a centrifugal mixer (ARV-310, Thinky Corp., Tokyo, Japan).

### 2.2. Preparation of Cellulose Nanopaper by Evaporation Drying

The conditioned dispersion (0.45 wt %, 22 g) was dropped evenly into a petri dish (9 cm diameter) silane-treated with decyltrimethoxysilane (KBM-3103, Shin-Etsu Chemical Co., Ltd., Tokyo, Japan) and was then dried at various temperatures (ranging from 45 °C to 85 °C) and relative humidities (ranging from 35% to 75%) in an environmental chamber (SH-642, ESPEC Corp., Osaka, Japan). After 30 min of pre-drying, the weight change of the dispersion was monitored using a balance every 2 min. After the weight remained constant at approximately 0.1 g (the weight of the nanopaper) and stopped changing for more than 30 min, the drying procedure was considered complete. The end of the drying time was considered the time at which the dried weight changed less than 0.045%.

### 2.3. Air Flow System in a Conventional Oven

The air flow system used in this work was a flat air nozzle (AFTCS15, MISUMI Group Inc., Tokyo, Japan) with 13 orifices of 9 mm diameter connected to an air compressor (PC3-5.5TL, YAEZAKI KŪATSU Co., Ltd., Tokyo, Japan). The system softly blew air (air flow rate: 0.4–0.5 L/min) toward the dispersion to remove the saturated water vapor at the water/air interface (see Figure S1). When the air flow system was blowing, the RH directly above the water/air interface was reduced, thus increasing the evaporation rate.

### 2.4. Characterization

The thickness of the obtained nanopaper was measured by a digital thickness gauge (G2-205M, Ozaki Mfg Co., Ltd., Tokyo, Japan). Total transmittance of the obtained nanopaper was measured by a UV-vis-NIR spectrophotometer (UV-3600 Plus, Shimadzu Corp., Kyoto, Japan). The haze of the obtained nanopaper with thickness of 10 ± 1 μm was measured by a haze meter (HZ-V3, Suga Test Instruments Co., Ltd., Tokyo, Japan).

X-ray diffraction patterns were recorded using a Rigaku MiniFlex600 (Tokyo, Japan) with Cu–Kα radiation and a scanning angle (2θ) range of 5–40° at 40 kV voltage and 15 mA current. The crystallinity index of cellulose I was calculated from the (200) reflection (2θ = ca. 22.6°) as previously described [14,27].

The surface roughness of the nanopaper was determined using an atomic force microscope (AFM, Nanocute, SII Nano Technology Inc., Chiba, Japan) in the dynamic force mode (measurement range: 10 μm × 10 μm).

For the density measurement, the obtained nanopaper sample (∼50 mg) was placed into weighing bottle and dried under vacuum at 105 °C for 3 h for further density analyzing. The weight of the sample was measured by an analytical balance with an accuracy of ±0.03 mg for 100 g (BM-252, A&D Co., Ltd., Tokyo, Japan). The density was analyzed using a BELPycno helium pycnometer (Bonsai Advanced Technologies SL., Madrid, Spain) [28].

## 3. Results and Discussion

**Figure 1:**
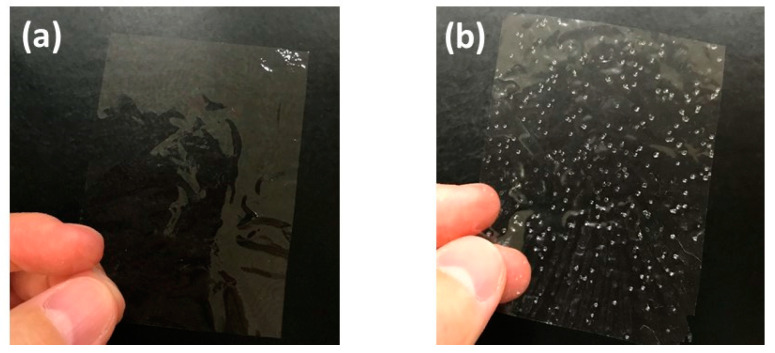
Appearance of transparent cellulose nanopaper prepared using evaporation drying at (**a**) 50 °C for 12 h and (**b**) 110 °C for 2 h.

In this study, we used 3–5-nm wide TEMPO-oxidized cellulose nanofibers. When such fine nanofibers are homogeneously dispersed in water, their concentration is usually adjusted to less than 1 wt % because of their high viscosity. Therefore, it took 12 h at 50 °C using evaporation drying to produce 10-µm-thick cellulose nanopaper (Figure 1a). The simplest way to reduce the drying time is to increase the drying temperature. In the extreme case, when the dispersion was heated at 110 °C, the nanopaper was produced in only 2 h. However, when the dispersion was heated at a temperature higher than the boiling point of water, the obtained nanopaper contained many air bubbles (Figure 1b). From these experiments, it was apparent that drying above the boiling point dramatically reduced the drying time but adversely affected the transparency.

**Figure 2:**
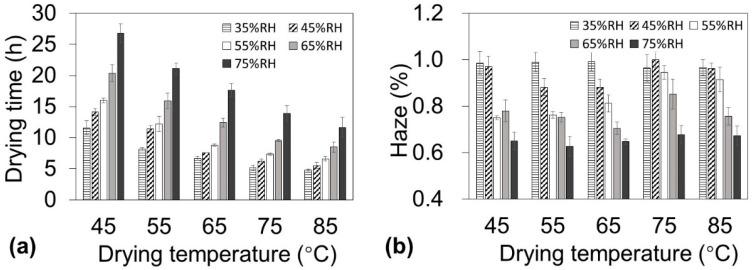
Drying time of nanofiber dispersions (0.45 wt %, 22 g) and haze of nanopaper with 10 ± 1 µm thickness under various temperature and humidity conditions. (**a**) Drying time and (**b**) haze as a function of drying temperature.

To produce low-haze cellulose nanopaper in a short drying time, the temperature and humidity must be carefully designed during the drying process. It is well known that for the same amount of water vapor when the atmospheric temperature is increased in a closed system, the absolute humidity remains constant but the RH decreases. Because the moisture evaporation is affected by not only the temperature but also the RH, the effect of temperature and humidity should be separately discussed when the drying schedule is determined. Therefore, the cellulose nanofiber dispersion was dried using an environmental chamber that enabled independent control of the ambient temperature (45–85 °C) and RH (35–75%). Although these two factors can both affect the drying time, the RH plays a more important role in determining the haze of the nanopaper. At each temperature, a lower RH reduced the drying time (Figure 2a). For example, at 65 °C, decreasing the RH from 75% to 35% reduced the drying time from 17.7 h to 6.7 h. In contrast, at each temperature, a lower RH increased the haze (Figure 2b). For example, at 65 ˚C, decreasing the RH from 75% to 35% increased the haze from 0.65% to 0.99%. These results indicate that a short drying time requires a lower RH, whereas lower haze requires a higher RH. Therefore, these contradictory humidity conditions must be taken into account to reduce both the drying time and haze.

**Figure 3:**
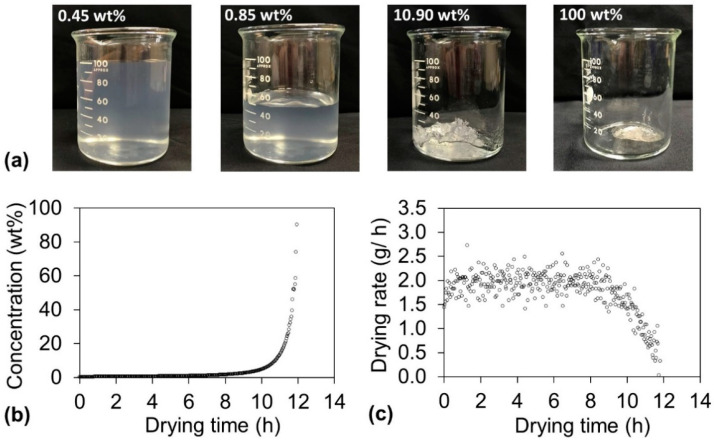
(**a**) Change of appearance of cellulose nanofiber dispersion during drying. The original dispersion was liquid-like at a concentration of 0.45 wt % and maintained the same appearance until 0.85 wt %. As the water evaporation proceeded, at approximately 10 wt %, the dispersion became paste-like and finally became a solid. (**b**) Dispersion concentration and (**c**) drying rate change as a function of drying time at 55 °C and 55% RH.

Before the contradictory humidity conditions required to achieve the optimal drying time and haze could be discussed, the drying behavior needed to be thoroughly understood. While drying the dispersions, their appearance drastically changed (Figure 3a). The sample before drying was liquid-like at a concentration of 0.45 wt %, and it maintained a liquid-like appearance until the concentration increased to 0.85 wt %. However, as the water evaporation proceeded, the sample became paste-like at approximately 10 wt % and finally became solid at approximately 100 wt %. These appearance changes have a close relationship with the concentration as well as the drying rate. The concentration change is plotted as a function of the drying time at 55 °C and 55% RH in Figure 3b. The concentration linearly increased when the sample was liquid-like (until approximately 8 h), whereas the concentration exponentially increased after the sample became paste-like (from approximately 8 h). These appearance changes also reflect the drying rate (Figure 3c). For the liquid-like state (until approximately 8 h), the water evaporation occurred at the sample surface because the surface was rich in water. Therefore, the drying rate was constant during this period, which is denoted as the constant-drying-rate period. When the sample turned paste-like (from approximately 8 h), mainly the internal water of the sample evaporated. The evaporation interface proceeded further inward to the sample body with extended drying time; therefore, the drying rate decreased, and this period is denoted the falling-drying-rate period. Table S1 summarizes the three drying times (total drying time, constant-drying-rate-period time, and falling-drying-rate-period time), the transit concentration from the constant-drying-rate period to the falling-drying-rate period, and the haze of the obtained cellulose nanopaper for all the drying conditions. For transparent cellulose nanopaper, haze is strongly dependent on the internal microstructure, more specifically, the packing of nanofibers with different density [7]. Therefore, the haze might be controlled in the falling-drying-rate period, during which the water evaporation occurred inside the nanopaper and affected the packing of nanofibers.

**Figure 4:**
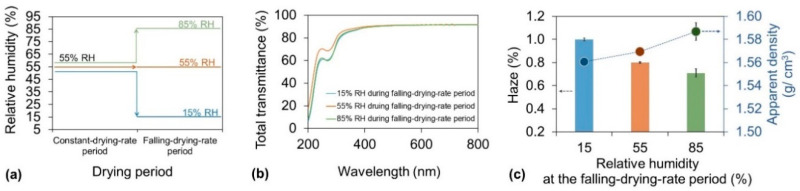
(**a**) Drying strategy for investigation of the relationship between the haze and RH during the falling-drying-rate period. During the drying process, the temperature was 55 °C for the entire period, and the RH was 55% during the constant-drying-rate period and 15%, 55%, or 85% during the falling-drying-rate period; (**b**) Total transmittance of cellulose nanopaper; (**c**) Corresponding haze (bar) and apparent density (dot) of cellulose nanopaper.

To overcome the contradictory humidity conditions required to optimize the drying time and haze of cellulose nanopaper, the effect of the RH on the haze during the falling-drying-rate period was evaluated. In these experiments, the drying temperature was set at a constant value of 55 °C while the RH was set at 55% during the constant-drying-rate period and 15%, 55%, or 85% during the falling-drying-rate period (Figure 4a). With the same high total transmittances (∼90%) in the visible wavelength range of 400–800 (Figure 4b) and similar high crystallinities (∼80%) (Figure S2), there exists an interesting relationship between the RH in the falling-drying-rate period and the haze of the nanopaper (Figure 4c bar). The lowest RH of 15% produced the worst haze of the nanopaper of 1.00%. The RH of 55% got the better haze of 0.80%, and the highest RH of 85% resulted in the best haze of 0.71%. The haze was caused by light scattering inside or at the surface of the transparent nanopaper. The surface roughness (Rq) of the resultant nanopaper was about 3.3 nm, 2.9 nm, and 3.1 nm, respectively. Therefore, the variations of the obtained haze values were mainly caused by the different porosities of nanopaper. The apparent density in these nanopaper showed a close relationship with the RH during the falling-drying-rate period (Figure 4c dot). When the RH increased in the falling-drying-rate period, the apparent densities in the nanopaper increased. These results suggest that a lower RH during the falling-drying-rate period accelerated water evaporation from inside the sample, and the inhomogeneous aggregation of the nanofibers during the quick fabrication resulted in the formation of more air voids inside the sample. However, a higher RH decelerated the water evaporation, which provided the slow formation of interactions between nanofibers, thus leading to less air voids remaining inside the sample. Therefore, to achieve low haze of the nanopaper, the RH should be higher during the falling-drying-rate period.

**Figure 5:**
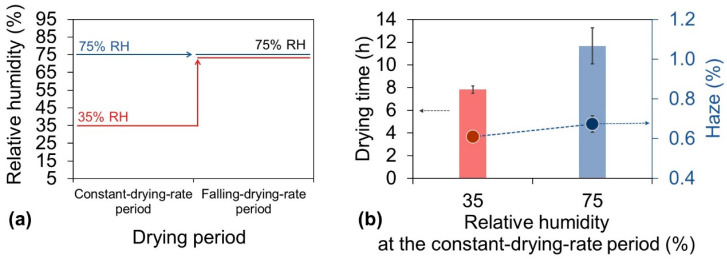
(**a**) Drying strategy for investigation of the relationship between the haze and RH during constant-drying-rate period. (**b**) Corresponding drying time (bar) and haze (dot) of cellulose nanopaper. During the drying process, the temperature was 85 °C for the entire period, and the RH was 35% or 75% during the constant-drying-rate period and 75% during the falling-drying-rate period.

After clearly understanding the humidity conditions needed to reduce the haze of the nanopaper, we attempted to determine the humidity conditions needed to reduce the drying time. Because the constant-drying-rate period accounts for 70–90% of the total drying time (Table S1), reducing the duration of this period is the most effective way to decrease the total drying time. The effect of the RH on the duration of the constant-drying-rate period was evaluated. In these experiments, the drying temperature was set at 85 °C for the entire drying time, and the RH was set at 35% or 75% during the constant-drying-rate period and 75% during the falling-drying-rate period (Figure 5a). When the RH remained at 75% during both periods, as in the traditional single-stage drying process, the drying time was 11.7 h (Figure 5b bar). However, when the new multi-stage drying process was applied with conditions of 35% RH during the constant-drying-rate period and 75% RH during the falling-drying-rate period (Figure 5a), the drying time was only 7.6 h, a decrease of 35% (Figure 5b bar). The RH during the different drying periods played different roles in determining the haze level. During the falling-drying-rate period, decreasing the RH led to deterioration of the haze of the cellulose nanopaper because the evaporation was mainly from the internal water of the film. In contrast, during the constant-drying-rate period, the water evaporation occurred mainly at the air/water interface of the dispersion. Therefore, even when the drying rate increased by applying a lower relative humidity (35%) during this period, the resulting cellulose nanopaper still maintained a low haze of 0.61%, which was comparable to the haze of 0.67% from that dried under a constant higher RH (75% in this case) with shorter drying time (Figure 5b dot). From these results and discussion, it was apparent that a lower RH reduced the drying time during the constant-drying-rate period when the evaporation was mainly from the water-rich interface, and the subsequent higher RH during the falling-drying-rate period reduced the haze of the cellulose nanopaper when the evaporated water mainly came from inside the sample. Therefore, this multi-stage drying process satisfies the contradictory humidity conditions required to reduce both the drying time and haze.

**Figure 6:**
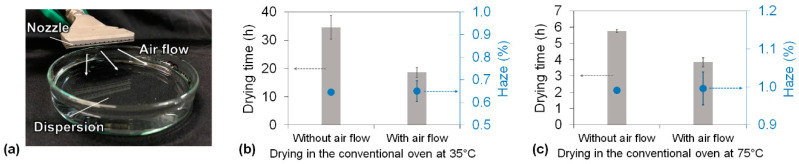
(**a**) The air flow system softly blew toward the dispersion to remove the water vapor at the water/air interface. Drying time of the cellulose nanofiber dispersion and haze of the cellulose nanopaper in the conventional oven with and without the air flow system under (**b**) 35 °C drying and (**c**) 75 °C drying.

To reduce the drying time of the dispersion and the haze of the cellulose nanopaper, we proposed a humidity-controlled multi-stage drying process using an environmental chamber that can independently control the ambient temperature and RH. However, in a conventional oven, only the temperature and not the humidity can be controlled, and the humidity inside the conventional oven is usually quite low. For example, the RH of a conventional oven is approximately 18% at 35 °C and 4% at 75 °C when the room temperature and RH are 20 °C and 70%, respectively. Therefore, achieving higher RH during the falling-drying-rate periods would be difficult using a conventional oven. Theoretically, the vapor pressure of the air vapor directly above the surface of the drying sample always remains approximately the saturated vapor pressure when water is evaporated from the sample. Air flow to the sample surface decreases the vapor pressure below the saturated vapor pressure, and air retention increases the vapor pressure back to the saturated vapor pressure. Therefore, we developed an air flow system in a conventional oven that enables control of the RH directly above the surface of the dispersion (Figure 6a, see also Figure S1). When the air flow was not applied at 35 °C drying, the saturated vapor pressure was maintained at the dispersion surface during all the drying periods. As a result, transparent cellulose nanopaper with 0.64% haze was produced in 36.6 h (Figure 6b). In contrast, when the air flow was applied only during the constant-drying-rate period at 35 °C drying, the vapor pressure of the dispersion surface became lower than the saturated vapor pressure, resulting in a faster drying rate and thus contributing to a shorter constant-drying-rate period; the saturated vapor pressure was maintained during the falling-drying-rate period to ensure dense packing. As a result, the total drying time decreased to 18.6 h while maintaining the haze of the transparent cellulose nanopaper at 0.65% (Figure 6b). Moreover, when the air flow was applied only during the constant-drying-rate period at 75 °C drying, the total drying time decreased from 5.7 h to 3.8 h while maintaining the haze of the transparent cellulose nanopaper at approximately 1% (Figure 6c). These results suggest that the air flow during the constant-drying-rate period increased the drying rate and that the air retention during the falling-drying-rate period did not produce more air voids than usual. Therefore, our concept of a humidity-controlled multi-stage drying process can be applied not only in an environmental chamber, where the humidity can be controlled, but also in a conventional oven, where the humidity cannot be controlled.

## 4. Conclusions

This work demonstrates the possibility of reducing the drying time of aqueous cellulose nanofiber dispersions while maintaining the low haze of the resulting cellulose nanopaper. When the dispersion was dried using an environmental chamber, the drying time and haze were observed to be more sensitive to the RH than to the temperature. However, reduction of the drying time and haze required contradictory humidity conditions. An increase in RH positively affected haze reduction but negatively affected the drying time. From monitoring the weight change of the dispersion, it appeared that the drying procedure consisted of a constant-drying-rate period and a falling-drying-rate period. The RH could be kept lower during the constant-drying-rate period, which was related less to the microstructure of the nanopaper and more to its drying time because water evaporation during this period mainly occurred at the water-rich surface. For the falling-drying-rate period, the evaporated water was mainly from internal water, and a relatively higher RH during this period promoted the tight packing of cellulose nanofibers, resulting in a lower haze. This humidity-controlled multi-stage drying process can also be applied in a conventional oven with the addition of a simple air flow system. Regardless of the oven type, the air above the surface of the sample always remained at the saturated vapor pressure, which inhibited the quick water evaporation. Therefore, when the air flow system was applied to the surface during the constant-drying-rate period, thus removing the water vapor above the surface, the total drying time was reduced to less than 4 h while maintaining the low haze. This humidity-controlled multi-stage drying process demonstrated the effectiveness in the nanopaper preparation, and has a great potential for the widespread use of nanopaper-based flexible electronics.

## Supplementary Materials

The following are available online at https://www.mdpi.com/2079-4991/10/11/2194/s1, Table of drying time (total drying time, constant-drying-rate-period time, and falling-drying-rate-period time), transit concentration (the dispersion transit from the constant-drying-rate period to falling-drying-rate period), and haze of obtained cellulose nanopaper under different drying conditions (Table S1); figure of the air flow system used to control the humidity in the conventional oven (Figure S1); figure of X-ray diffraction profiles of nanopaper dried under different conditions (Figure S2).
