# Supplementary material for: High-Speed Fabrication of Clear Transparent Cellulose Nanopaper by Applying Humidity-Controlled Multi-Stage Drying Method
Source: Nanomaterials (Basel). 2020 Nov 4;10(11):2194. doi: 10.3390/nano10112194 (PMC7693990; doi:10.3390/nano10112194)
Supplement: Supplementary file 1 [file nanomaterials-10-02194-s001.pdf]

nanomaterials

High-Speed Fabrication of Clear Transparent Cellulose Nanopaper by Applying Humidity-Controlled Multi-Stage Drying Method

Chenyang Li [1], Takaaki Kasuga [1], Kojiro Uetani [1], Hirotaka Koga [1] and Masaya Nogi [1,*]

[1] The Institute of Scientific and Industrial Research, Osaka University, 8-1 Mihogaoka, Ibaraki, Osaka 567-0047, Japan; lichenyang@eco.sanken.osaka-u.ac.jp (C.L.); tkasuga@eco.sanken.osaka-u.ac.jp (T.K.); uetani@eco.sanken.osaka-u.ac.jp (K.U.); hkoga@eco.sanken.osaka-u.ac.jp (H.K.)

* Correspondence: nogi@eco.sanken.osaka-u.ac.jp; Tel.: +81-6-6879-8441 (M.N.)

Table S1. Drying time, transit concentration (the dispersion transit from constant drying rate period to falling drying rate period), and haze of obtained cellulose nanopaper. The drying times listed include the total drying time, constant-drying-rate-period time, and falling-drying-rate-period time. The relative humidity and temperature were constant in all the periods under all the conditions.

Relative humidity	Temperature (°C)	Total drying time (h)	Constant drying rate period		Falling drying rate period		Transit concentration (wt %)	Haze (%)
			Period time (h)	Ratio (%)	Period time (h)	Ratio (%)		
RH 35%	45	12.8	9.3	73	3.5	27	2.45	1.00
RH 35%	55	8	5.7	71	2.3	29	2.32	1.02
RH 35%	65	6.3	4.8	76	1.5	24	2.52	1.00
RH 35%	75	5.3	3.7	70	1.6	30	2.46	0.96
RH 35%	85	4.5	3.3	73	1.2	27	2.38	0.99

Relative humidity	Temperature (°C)	Total drying time (h)	Constant drying rate period		Falling drying rate period		Transit concentration (wt %)	Haze (%)
			Period time (h)	Ratio (%)	Period time (h)	Ratio (%)		
RH 45%	45	14.6	11.6	79	3.0	21	3.96	0.94
RH 45%	55	11.7	8.4	72	3.3	28	3.72	0.85
RH 45%	65	7.6	5.9	78	1.5	22	3.34	0.91
RH 45%	75	5.8	4.7	81	1.6	19	3.97	0.98
RH 45%	85	5	3.9	78	1.2	22	4.35	0.95

Relative humidity	Temperature (°C)	Total drying time (h)	Constant drying rate period		Falling drying rate period		Transit concentration (wt %)	Haze (%)
			Period time (h)	Ratio (%)	Period time (h)	Ratio (%)		
RH 55%	45	16.3	13.9	85	2.4	15	4.27	0.75
RH 55%	55	12.1	10.4	86	1.7	14	4.08	0.76
RH 55%	65	8.6	6.8	79	1.8	21	4.07	0.78
RH 55%	75	7.1	5.8	82	1.3	18	3.46	0.93
RH 55%	85	4.5	3.3	73	1.2	27	2.38	0.91

Relative humidity	Temperature (°C)		Constant drying rate period	Falling drying rate period	Transit concentration	Haze (%)

		Total drying time (h)	Period time (h)	Ratio (%)	Period time (h)	Ratio (%)	(wt %)	
RH 65%	45	18.9	15.9	84	3.0	16	4.6	0.82
RH 65%	55	14.5	12.7	88	1.8	12	4.75	0.78
RH 65%	65	12.9	11.0	85	1.9	15	4.72	0.73
RH 65%	75	9.7	7.9	81	1.8	19	4.06	0.88
RH 65%	85	8	6.5	81	1.5	19	4.61	0.74

Relative humidity	Temperature (°C)	Total drying time (h)	Constant drying rate period		Falling drying rate period		Transit concentration (wt %)	Haze (%)
			Period time (h)	Ratio (%)	Period time (h)	Ratio (%)		
RH 75%	45	25.2	23.7	94	1.5	6	9.33	0.61
RH 75%	55	20.4	18.9	93	1.5	7	8.72	0.67
RH 75%	65	17.5	15.3	87	2.2	13	6.98	0.64
RH 75%	75	12.8	11.5	90	1.3	10	7.28	0.72
RH 75%	85	11.3	10.1	89	1.2	11	7.82	0.67

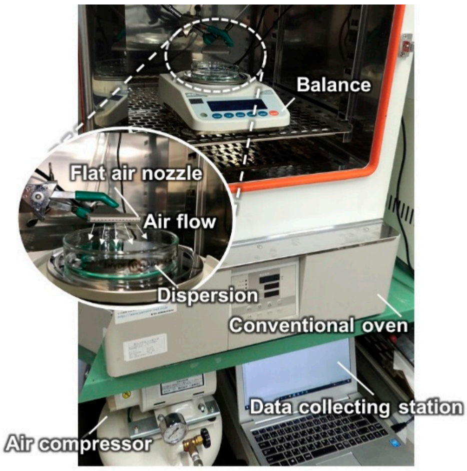

Figure S1. The air-flow system used to control the humidity in the conventional oven. The air-flow system was a flat air nozzle with 13 orifices of 9 mm diameter connected to an air compressor. The system softly blew air (air flow rate: 0.4-0.5 L/min) toward the dispersion to remove the saturated water vapor at the water/air interface. When the air-flow system was blowing, the RH directly above the water/air interface was reduced, thus increasing the evaporation rate.

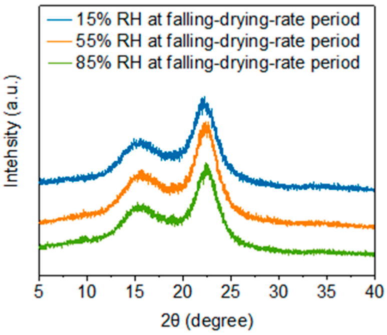

Figure S2. X-ray diffraction profiles of nanopaper dried under different conditions. During the drying process, the temperature was 55 °C for the entire period, and the RH was 55% during the constant-drying-rate period and 15%, 55%, or 85% during the falling-drying-rate period.
